# Active commuting to school in Finland, the potential for physical activity increase in different seasons

**DOI:** 10.3402/ijch.v75.33319

**Published:** 2016-12-05

**Authors:** Jouni Kallio, Salla Turpeinen, Harto Hakonen, Tuija Tammelin

**Affiliations:** LIKES – Research Center for Sport and Health Sciences, Jyväskylä, Finland

**Keywords:** active travel, winter, children, travel mode, cost-effect

## Abstract

**Background:**

Active commuting to school (ACS) can be a significant source of physical activity and provide many health benefits.

**Objective:**

This study identified the potential to increase physical activity levels by promoting ACS in Finnish schools and evaluated the effects of season, distance and age on ACS.

**Design:**

Data were collected with a questionnaire from 5,107 students, aged 10–16, in 45 comprehensive schools in Finland. The distance and the mode of transport to school in different seasons were self-reported.

**Results:**

The prevalence of ACS was over 80% during spring/fall for those living 0–5 km from school. ACS was inversely associated with the distance to school and was lower in winter compared to spring and fall. Cycling is less common in winter, especially among girls and younger students. The potential for increasing students’ physical activity levels via ACS seems to be largest in winter, especially among students living 1–5 km from school. The variation in the prevalence of ACS between schools was large, especially in winter.

**Conclusions:**

When planning interventions to promote ACS, one is encouraged to acknowledge and evaluate the potential in the selected target schools in different seasons. The potential varies largely between schools and seasons and is highly dependent on students’ commuting distances.

Active commuting to school (ACS) can be a significant source of physical activity (PA). From the 49 studies analysed in a recent review by Larouche et al. (1), 40 showed an association between ACS and higher PA levels. This association was observed in studies utilizing questionnaires, but also in most accelerometer and pedometer studies, despite their inability to measure PA in cycling (2). The positive effect of ACS on daily PA levels is supported by the evidence from studies that have measured PA separately on weekends, because a higher PA level on active commuters has only been found during weekdays (3–5). Among students living in urban environments, more than half of the daily moderate to vigorous PA has been found to occur while commuting (6).

Several studies have found active school commuting to be associated with better fitness and a more favourable body composition (1). The evidence on this association is stronger for cycling than for walking for both fitness (7,8) and weight (9). There may also be an association between ACS and lower cardiovascular risk (10), especially in cyclists (11).

The prevalence of active commute varies by country and by culture. In the Global Matrix of the Report Cards on Physical Activity for Children and Youth (12), the proportion of active school commuters for distances below 3 km ranged from less than 15% in the United States (13) to 75% in Finland. Across countries, distance to school seems to be the most important factor determining the popularity of ACS (14–20). Boys have been found to commute actively more commonly than girls (18,21) and bike more often (14,22,23). The most active age for active commuting seems to be between 9 and 13 years of age (15,19,24,25).

There are only few studies that have examined the effects of seasons on ACS, especially in colder climates like the Nordic countries. Previous Norwegian and Canadian studies have not found large seasonal differences in ACS (23,26–28). However, walking seems to be more common and cycling less common in winter compared to spring, summer or fall (23,26).

Interventions focusing on promoting ACS have been very heterogeneous in both design and results. The improvements have ranged between 3 and 64% and, although distance is the most important factor determining the prevalence of ACS, targeting interventions based on distance seems to be very rare (29). When directing funds to measures intended to increase children’s PA, it is important to know whom to target and where the greatest potential is for change. Therefore, the aim of this study was to identify the potential to increase PA levels by promoting ACS in Finnish comprehensive schools and to evaluate the combined effects of distance, age, season and school on the prevalence of ACS.

## Methods

### Study population

This study was conducted in the spring of 2013 as a part of a larger study regarding the national Finnish Schools on the Move program (30). The participants were from 45 primary and lower secondary schools, representing all regions of Finland. Of the schools, 40 had just begun in the program. In total, 5,107 students (2,592 girls, 2,515 boys) from grades 4 to 9 (aged 10 to 16) participated in the study.

Age, gender, distance to school and the mode of transport to school were self-reported through a web-based questionnaire that was completed in class. Of the 8,273 students in the participating schools, 5,107 agreed to complete the questionnaire, giving a total participation rate of 62% (Table I). Students from 36 schools (4,156 students) responded anonymously, and no individual identification information was collected. Students from nine schools (951 students) participated in a more detailed follow-up study that required individual identification and a written consent from both the students and their guardians. The study protocol was approved by the Ethics Committee of the University of Jyväskylä.

**Table I:** Description of the study population

	Grades 4–6			Grades 7–9			Total		
			
	Boys	Girls	Total	Boys	Girls	Total	Boys	Girls	Total
	
N	1,497	1,483	2,980	1,018	1,109	2,127	2,515	2,592	5,107
Age (mean +SD)	11.3+1.0	11.3+1.0	11.3+1.0	14.1+1.0	14.1+1.0	14.1+1.0	12.4+1.7	12.5+1.7	12.5+1.7
Distance to school									
0–1 km	49.9%	49.1%	49.5%	32.1%	29.8%	30.9%	42.7%	40.8%	41.8%
1.1–3 km	31.2%	33.1%	32.1%	26.7%	27.6%	27.2%	29.4%	30.8%	30.1%
3.1–5 km	8.2%	8.1%	8.2%	13.6%	14.4%	14.0%	10.4%	10.8%	10.6%
>5 km	10.7%	9.7%	10.2%	27.7%	28.2%	27.9%	17.5%	17.6%	17.6%

### Distance to school

Participants were asked, ‘How long is your distance to school?’. The response alternatives were: (a) less than 500 m, (b) 500 m–1 km, (c) 1.1–2 km, (d) 2.1–3 km, (e) 3.1–5 km and (f) more than 5 km. If the distance was more than 5 km, students were also asked to specify the actual distance in kilometres.

### Mode of commuting to school

The mode of commuting to school was assessed with the following question: ‘How do you generally commute to and from school? Choose the most common mode of commuting’. The response alternatives were (a) walking, (b) cycling, (c) by parent’s car, (d) by school transit and (e) by other motorized vehicle.

Finland has four seasons, and the temperatures are quite different in the winter (average −8.6°C) compared to summer (13.6°C), fall (+2.6°C) or spring (+1.2°C). For this reason, the previous question was answered separately for the situation: (a) in winter and (b) in spring and fall. The answers regarding the most common mode of transport were grouped to active commuting (walking and cycling) and passive commuting (all modes of motorized transportation).

In order to estimate the population most likely to benefit from interventions, two calculations were made. Firstly, the student population living within 5 km from school was divided into three groups based on the commuting distance (0–1.0, 1.1–3.0 and 3.1–5.0 km). The students living more than 5 km from school were excluded from this analysis, as the prevalence of ACT was relatively low (<20%) and the students are entitled by law to free transportation by the municipality. The proportion of passive commuters was then calculated for each group and season. This proportion was calculated in relation to the whole population so that the largest target groups for ACT could be identified. Secondly, in order to estimate the potential of school commute interventions, the amount of PA that could be increased by activating the students that were passive commuters was estimated for each group. The potential addition of daily PA for passive commuters was calculated by dividing the distance to school and back by the average commuting velocity of the student’s age group. In the calculations, the velocities for walking were estimated to be 4 km/h for fourth to sixth graders and 5 km/h for seventh to ninth graders based on a study by Whittle (31) and adjusting the estimates for two age groups and the speed-reducing effects of traffic in the students’ urban neighbourhoods. The cycling velocity was estimated to be 10 km/h after adjusting the previously reported 13 km/h (32) for the urban commute. Walking velocity was not adjusted, as it was considered less likely to be affected by the environment.

Variation in the prevalence of ACS between schools was assessed by comparing the results in 10 schools with at least 30 study participants living 1.1–2.0 km from school. This range of distance was chosen as an example of a commute distance long enough for potential physiological benefits, but not too long for walking or cycling. These analyses included a total of 458 students.

### Statistics

The prevalences of ACS during winter and spring/fall were calculated by commuting distance, age and gender. The separate prevalences of cycling and walking to school were similarly calculated. Using SPSS for Windows, version 20 (SPSS Inc., Chicago, IL), the differences in ACS between seasons were examined with McNemar’s test. The associations between socio-demographic variables (distance, gender and primary vs. lower secondary school) and ACS were tested for significance using the Pearson’s chi-square.

## Results

The average distance to school was longer among older students compared to younger students as described in Table I. The commuting distance was no more than 1 km for 50% of the younger students and for 31% of the older students. Furthermore, the commuting distance was more than 5 km for 10 and 28% of the younger and older students, respectively.

**Fig. 1:**
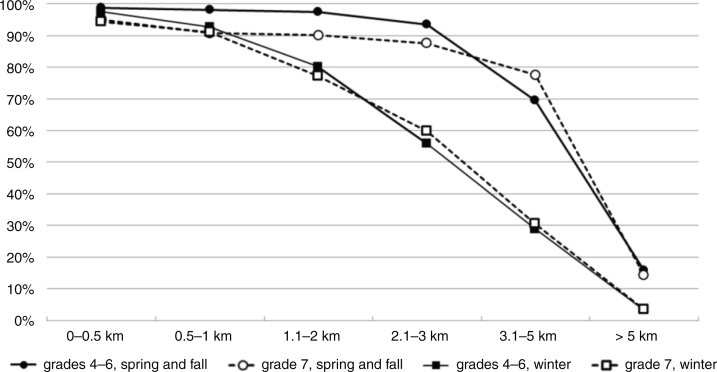
The prevalence of students commuting actively (walking or cycling combined) to school (%) according to the distance to school. Results are presented separately for different seasons and age groups: spring/fall (circle) and winter (square), grades 4–6 (single line) and grades 7–9 (dashed line).

In general, ACS was largely related to distance to school and season in both genders and age groups (Fig. 1). The prevalence of ACS was inversely associated with the distance of commuting to school (p<0.001). In the spring and fall, almost all the children (95%) walked or cycled to school when the distance was less than 3 km, with students in primary school being more active than students in lower secondary school (97% vs. 91%, p<0.001). Among students living 3–5 km from school, the physically active forms of commuting were less common, and the age effect was reversed, as 70% primary school students and 78% of lower secondary school students were active commuters (p<0.005). The prevalence of ACS was the lowest among students living more than 5 km from school, as only 16% of primary school and 14% of lower secondary school students were active commuters in this group.

The prevalence of active commuting was lower in winter compared to spring and fall in both age groups (Fig. 1). On average, in spring and fall, 79% of students were commuting actively, whereas in winter the prevalence of ACS was 63%. The difference in ACS between seasons was greatest between distances of 2–5 km.

No consistent gender differences were observed in the prevalence of active school commute in either seasons or age groups. In spring and fall, 92% of the boys and 93% of the girls living within 5 km from school were active commuters. In the winter, the prevalence for these students was 79% for boys and 75% for girls.

**Fig. 2:**
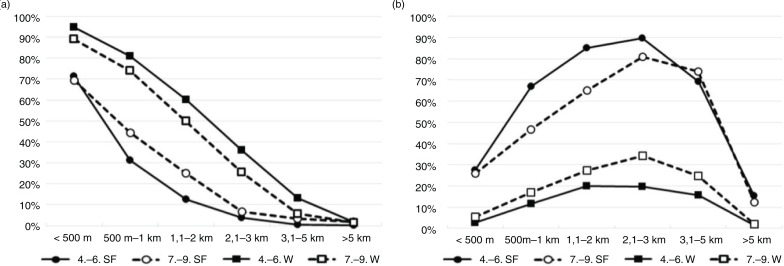
The prevalence of students walking (A) or cycling (B) to school according to the distance to school. Results are presented separately for different seasons and age groups: winter (W; squares) and spring and fall (SF; circles) months for grades 4–6 (single line) and 7–9 (dashed line).

When walking and cycling were evaluated separately (Fig. 2), walking to school was found to be more common during winter (50%) compared to spring and fall (25%; Fig. 2a). In contrast, cycling was more common during spring and fall (54%) compared to winter (13%; Fig. 2b). In spring and fall, older students walked to school more often (p<0.01) and during winter, cycled to school more commonly (p<0.001) than younger students. The prevalence of cycling to school was highest among students with a commuting distance of 2.1–3.0 km. Girls were more likely than boys to walk to school, whereas cycling to school was more common among boys. The gender differences were statistically significant in spring and fall for distances up to 1 km (p=0.00–0.02) and in winter for distances up to 3 km (p=0.00–0.02).

**Fig. 3:**
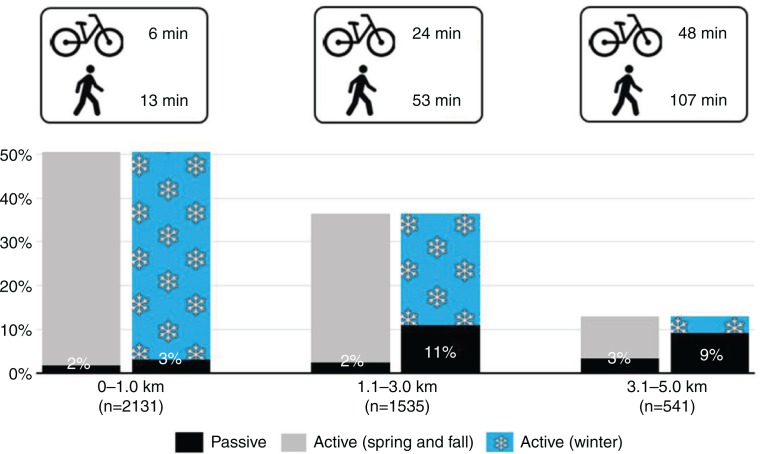
The potential targets and gains for interventions to promote physically active commuting to school in relation to the whole student population. The total height of the bar represents the proportion of the student population living 0–1.0 km, 1.1–2.0 km and 3.1–5.0 km from school. The values on the black bars indicate the proportion of passive commuters (expressed as % of whole student population). Next to the bicycle and walker symbols are the minutes of daily physical activity that would be added for both transportation modes and for each distance (calculated as an average of each range).

The potential to increase PA among students by getting passive commuters to walk or cycle to school is described in Fig. 3. As the green (spring and fall) and blue (winter) bars indicate, 50% of the students in this study lived within 1 km from school, 37% of the students lived 1.1–3.0 km and only 13% of the students lived 3.1–5.0 km from school. This means that although passive commuting (black bars) is most common among students living furthest from school, the absolute number of passive commuters may not be much greater compared to the students with shorter commutes. As the black bars indicate, in spring and fall, only 7% of the whole student population commutes passively (2–3% in each distance group). Thus, the greatest potential target population (meaning the highest number of students) for increasing students’ PA by getting the passive commuters to walk or cycle to school was in winter among students living either 1.1–3.0 km (11% of the entire population) or 3.1–5.0 km (9% of the entire population) from school. The estimations of this potential as physically active minutes can be seen in the top part of Fig. 3. The students living 1.1–3.0 km from school would increase their daily activity on average by 53 min if they were to walk to school and by 24 min if they were to use their bikes. The corresponding increases in PA for the students living 3.1–5.0 km from school would be on average 107 min by walking and 48 min by cycling.

**Fig. 4:**
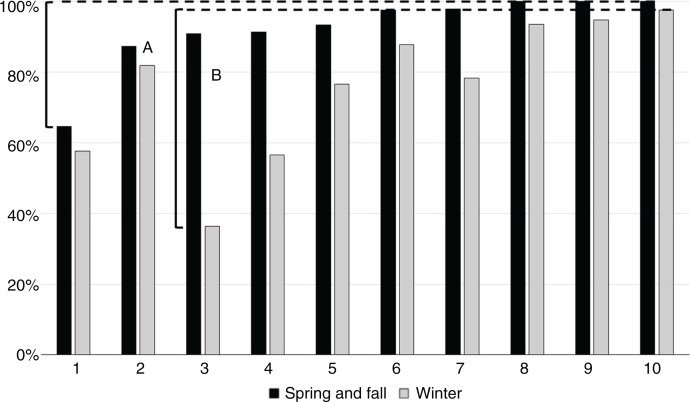
The prevalence of active commute to school in 10 suburban schools in different seasons for students living 1–2 km from school. Differences between schools with the highest and lowest ACS prevalences in spring/fall (A) and winter (B).

There were large differences between schools in the prevalence of ACS for students living 1.1–2.0 km from school, especially in winter (Fig. 4). The range in the percentage of active commuters varied from 65 to 100% in spring and fall (A), and from 36 to 98% in winter (B). The effects of winter on interschool variance was most visible in schools 3, 4 and 5; in spring and fall, these three schools were within 2% points, whereas in winter, the range was 41% points (C).

## Discussion

The prevalence of ACS was inversely associated with the distance to school and was lower in winter compared to spring and fall for students living 2–5 km from school. Walking was more common than cycling during winter months. The potential for increasing students’ PA levels by getting the passive commuters to walk or cycle to school was largest in winter, especially among students living 1.1–5.0 km from school. The variation in the prevalence of ACS between schools was large, especially in winter.

The effect of season on the popularity of ACS varied between commuting distances, travel modes and schools. To our knowledge, this is the first study investigating the combined effects of season and commuting distance on the prevalence of ACS. Among the students living within 1 km from school, the prevalence of ACS did not differ between seasons, as 97% of these students were also commuting actively in winter. In contrast, among students living 2–5 km from school, active commuting was decreased in winter by almost 50% (82% in spring/fall, 44% in winter). Almost all students living more than 5 km from school were passive commuters year round. This is understandable, as active commute beyond 5 km is quite time-consuming and free transportation is offered to these students by the municipality. In general, students, regardless of commuting distance, age or gender, were more likely to cycle in spring and fall and walk in winter. Assuming that none of the students cycled only in winter, 75% of all spring and fall cyclists switched to either walking or motorized transport in winter. This change was most visible in commuting distances of 1–5 km (Fig. 2b). The time people are willing to spend on active commuting has been found to be quite fixed (33). Because cycling speed is generally more than twice the speed of walking, a change in active transport mode from cycling to walking can increase the commute time by more than 100%. The absolute increase in commute time depends on distance and may be anything from a few minutes to more than an hour. This may explain why the passivating effect of winter is much larger in long, compared to short, commuting distances. Promotion of safe winter cycling in reasonable weather and with proper gear (e.g. studded tires) may thus be a potential way to improve PA and health in students, especially as the evidence on the health effects of ACS seem to be strongest for cycling (1).

The observed 15% seasonal difference in average ACS in Finland was larger compared to the previous 3–8% differences in Norwegian students (23,26) and the lack of any difference in Canadian students (27,28). A possible explanation for this could be that only Børrestad et al. (23) reported a similarly high proportion of spring and fall cyclists. In other studies, cycling was either much less common or not reported at all. Contrary to previous studies (15,18,19,21,24,25), age and gender were not significant determinants of overall prevalence of ACS when the results were evaluated taking into account the distance to school. However, cycling was more common among boys and middle-school students in winter. It may be that parents view winter cycling as risky and may be more protective of younger children and girls (34).

Although Finnish students are on average very active commuters in international comparison (12), there was variation between schools, especially in winter. A previous study by Robertson-Wilson et al. (27) found even greater variation in Canadian schools, ranging from 12 to 77%. This larger range may be due to a more heterogeneous study population that included schools in both urban and rural areas, whereas all the schools included in our analysis were suburban. The current findings highlight the importance of knowledge about individual schools before designing ACS interventions. We observed schools that have great potential to increase PA through the increase of ACS, while other schools may already be almost 100% active in spring and fall and some even all year round.

Choosing the most cost-effective PA interventions maximizes the total benefits of limited resources (35). The aim of this study was not to investigate the costs or effects of ongoing interventions. Instead, we analysed the current state of ACS in different student sub-populations in order to find the most potential targets for future programs. The effect of a PA intervention is often calculated as the total amount of activity that has been generated by the program. The maximal potential in increasing PA through promotion of ACS was therefore measured by multiplying the number of students currently commuting passively by the amount of time (daily) each of those students would gain via ACS. From Fig. 3, we can see that the greatest number of passive commuters could be targeted in winter among students living more than 1 km from school. The highest amount of PA could be gained among students living 3–5 km from school. However, a 1 h 47 min daily walk may be a hard for promoters to sell. Thus, it is more likely either to get the students living 1–3 km from school to walk 53 min or, if the weather allows, to cycle for 24 min daily. Adding PA by promoting ACS is important in winter because children have been shown to be less active in that season, particularly in regions that experience long, cold winters (36–38).

The main limitations of this study are related to the questionnaire used for data collection. Measuring PA in children is difficult, and both direct and indirect methods have limitations (39). Utilizing accelerometry would eliminate some of the errors of questionnaires. However, accelerometry has been shown to significantly underestimate the PA during cycling (40). Only the dominant mode of transportation was asked, which does not allow combinations like walking to the bus stop. Some students have divorced parents and may walk to school from one parent’s house and take the bus from the other. It is also possible that the travel mode varies within a season. Recording daily travel modes would require a journal, which in a study with more than 5,000 students would be an enormous task. Most of the schools (40 of 45) in the study had recently enrolled in the Finnish Schools on the Move program. However, we do not think that this affected the results, as the five schools that had not yet enrolled in the program had, on average, more active commuters. The schools in the study were from different parts of Finland and the commuting distance (72% within 3 km from school) was quite comparable to the national average (69% within 3 km from school) (41).

## Conclusions

Although active commute to school seems to be common among Finnish students in grades 4–7, there appears to be potential to increase the daily activity by promoting active forms of transportation to school. The greatest potential for activation in the spring and fall is for students who live more than 3 km from school. For the winter season, active commuting begins to decline at shorter distances. Because the transportation mode varies largely between schools and seasons, one is encouraged to acknowledge and evaluate the potential in the selected target schools in different seasons when planning interventions to promote ACS.

## Conflict of interest and funding

This research has received funding from the Ministry of Education and Culture and from the Juho Vainio foundation.
